# Investigating older adults users’ willingness to adopt wearable devices by integrating the technology acceptance model (UTAUT2) and the Technology Readiness Index theory

**DOI:** 10.3389/fpubh.2024.1449594

**Published:** 2024-09-25

**Authors:** Chengzhen Wu, Gyoo Gun Lim

**Affiliations:** International Center for Electronic Commerce, School of Business, Hanyang University, Seoul, Republic of Korea

**Keywords:** behavioral intention, Unified Theory of Acceptance and Use of Technology 2, Technology Readiness Index, smart wearable devices, digital health literacy

## Abstract

**Objective:**

With the continuous advancement of wearable technology, smart wearable devices are increasingly recognized for their value in health monitoring, assessment, and intervention for the older adults, thus promoting intelligent older adults care. This study, based on the theoretical framework of the Unified Theory of Acceptance and Use of Technology 2 (UTAUT2) and the Technology Readiness Index (TRI) model, aims to identify and explore the key factors influencing older adults consumers’ willingness to adopt smart wearable devices and their impact mechanisms.

**Method:**

A questionnaire survey was conducted to collect valid data from 389 older adults respondents. Empirical analysis validated the model’s applicability and explored the key factors influencing acceptance.

**Results:**

Factors influencing the use of smart wearable devices by the older adults include performance expectancy (*β* = 0.152, *p* < 0.001), effort expectancy (*β* = 0.154, *p* < 0.001), social influence (*β* = 0.135, *p* < 0.05), facilitating conditions (*β* = 0.126, *p* < 0.05), hedonic motivation (*β* = 0.166, *p* < 0.001), price value (*β* = 0.182, *p* < 0.001), and digital health literacy (*β* = 0.189, *p* < 0.001). Additionally, optimism (*β* = 0.208, *p* < 0.001), innovativeness (*β* = 0.218, *p* < 0.001), and discomfort (*β* = −0.245, *p* < 0.001) significantly positively influenced performance expectancy, while optimism (*β* = 0.282, *p* < 0.001), innovativeness (*β* = 0.144, *p* < 0.01), discomfort (*β* = −0.239, *p* < 0.001), and insecurity (*β* = −0.117, *p* < 0.05) significantly positively influenced effort expectancy. Insecurity did not significantly influence performance expectancy. Performance expectancy and effort expectancy partially mediated the relationship between personality traits (optimism, innovativeness, discomfort, and insecurity) and behavioral intention. Digital health literacy significantly negatively moderated the relationship between performance expectancy and behavioral intention, as well as between effort expectancy and behavioral intention.

**Discussion:**

The study confirms that integrating the UTAUT2 model and TRI theory effectively explains the acceptance of smart wearable devices among older adults consumers, emphasizing the importance of enhancing digital health literacy in the design and promotion of smart health devices. The findings provide guidance for developers, increasing the acceptance and usage rate of these devices among the older adults.

## 1 Introduction

The “China Population Forecast Report 2023” indicates that by around 2032, the proportion of China’s older adults population aged 65 and above is expected to exceed 20% of the total population (1). Compared to most countries globally, China’s population age structure transitioned from an adult-oriented to an older adults-oriented structure in approximately 18 years, a process that typically takes 60–100 years in other countries. This rapid aging process brings urgency to China’s efforts to address aging issues, lacking sufficient buffer time to prepare comprehensive response strategies. When China entered an aging society in 2000, its *per capita* GDP was only half of the world average, far below that of developed countries (2). This economic level has resulted in most Chinese older adults lacking sufficient personal wealth for retirement. The immaturity of China’s capital markets and the lack of financial literacy among the older adults have left some older adults individuals without effective awareness and means for retirement. Against this socio-economic background, China’s aging problem is characterized by a large scale, rapid speed, and high complexity. In particular, the rising number of chronic disease patients will increase the demand for medical resources and the burden of medical expenses. Currently, demand for older adults care services in China far outstrips supply, imposing substantial pressure on the older adults (102). With this context in mind, the use of smart wearable device technology offers potential innovative solutions for tackling the problems of aging effectively. These devices, crucial for collecting health information, hold significant value in the medical health field. Smart wearable devices, also known as wearable computers, refer to electronic devices such as smartwatches, smart glasses, and smart clothing that can be worn on the body (3). They can provide multiple functions such as physical activity assessment, fall and fatigue prediction and detection, gait analysis, stability assessment, and sleep analysis. This array of functionality offers invaluable support for medical health services in local jurisdictions (4, 5). For instance, cuffless blood pressure technology can accurately monitor nighttime hypertension (6), and smartwatch applications can detect irregular pulses (7). Smart wearable devices are seen as a new technology to assist in older adults care and alleviate the increased social support costs associated with aging (8). The application of smart wearable devices helps improve the efficiency of doctor-patient communication, reduce patient discomfort, and enhance the quality of medical services. These devices can collect health data from the older adults in real time, providing doctors with accurate diagnostic information. They can also respond quickly and offer necessary assistance in emergencies. Moreover, through data analysis, smart devices can predict changes in the health status of the older adults, enabling early intervention and reducing medical costs. In summary, the development of smart wearable devices represents technological progress and serves as a crucial strategy for addressing the challenges of aging. These devices can offer personalized health management solutions for the older adults, enhance their quality of life, and reduce the burden on families and society. Therefore, promoting the use of smart wearable devices is highly desirable, especially considering China’s aforementioned demographic specifics. This study focuses on commercially available, non-customized everyday smart wearable devices. These devices are increasingly incorporating medical functionalities and capturing the market through third-party applications and promotional strategies. In the 2023 Chinese market, five major manufacturers—Apple, Huawei, Samsung, Xiaomi, and Imagine Marketing—hold over half of the market share (9). Despite the growth in older adults care services in China, there are still significant issues such as insufficient inclusive care services and lack of supporting infrastructure, leading most older adults individuals to prefer traditional home-based care, particularly in economically constrained rural areas. Consequently, high-priced customized devices are less popular, and the older adults show a preference for affordable non-customized devices. This study evaluates these devices to identify obstacles encountered in promoting them to older adults users, aiming to provide insights for industry development.

Although smart wearable devices offer many benefits for the health management of the older adults, their acceptance among older adults users remains low (10). This phenomenon is influenced by a complex interplay of social, psychological, and technological adaptation challenges. For instance, technological care and learning ability are critical factors in the older adult’s adoption of smart technology (11). In China, older adults individuals typically rely on family members for assistance with digital devices. Their resistance to change and new experiences, along with self-doubt about their learning abilities, contributes to low digital literacy levels (12). In rural areas and among low-income older adults groups, the prevalence of online fraud underscores the urgent need for improved online security and risk mitigation strategies. Limited understanding of internet technology, inadequate proficiency with smart devices, and restricted access to digital literacy resources exacerbate their reluctance (13). The implementation of Internet of Things (IoT) technology also brings safety and privacy concerns for the older adults. Due to generally low technological proficiency, older adults individuals are more vulnerable to online threats, further diminishing their willingness to adopt new technologies (14). Economic factors also present significant barriers to technology adoption. The low-income older adults population in China may struggle to afford the initial purchase cost of digital devices and the ongoing expenses associated with internet use (12). The factors mentioned above and their interrelationships underscore the complexity of technology adoption within the older adults demographic in China. Additionally, it is crucial to examine theoretical frameworks suitable for analyzing older adults users’ acceptance intentions. To better understand older adults users’ behavioral intentions, researchers have explored various theoretical frameworks, such as the Theory of Planned Behavior (15), the Unified Theory of Acceptance and Use of Technology (16), Diffusion of Innovations Theory (17), and the Expectation-Confirmation Model (3). Furthermore, there is ongoing work on the development and design improvements of smart wearable technologies, such as by Douhi et al. (18) and Lin et al. (19). Current research on smart wearable devices mainly focuses on younger users, lacking systematic in-depth analysis of older adults users’ intentions. While these theories provide important perspectives on analyzing technological acceptance behaviors, their applicability and effectiveness in the older adults population need further verification. In contrast, the UTAUT model proposed by Venkatesh et al. (20) and its extended version, the UTAUT2 model (21), have shown strong explanatory power in various fields. The UTAUT2 model can be applied across different domains and effectively explains the process of consumers’ acceptance and adoption of new technologies (22). This study employs the UTAUT2 model to investigate the factors influencing older adults users’ adoption of smart wearable devices. Specifically, we examine performance expectancy (how older adults users perceive the usefulness of smart wearable devices in improving health management and overall quality of life), effort expectancy (the ease with which the older adults can use smart wearable devices), social influence (the impact of social networks, such as family and friends, on the older adult’s decision to use smart wearable devices), facilitating conditions (the availability of resources and support necessary to use smart wearable devices), hedonic motivation (whether the enjoyment or pleasure derived from using smart wearable devices encourages their adoption by the older adults), and price value (an economic assessment from the perspective of the older adults, evaluating whether they find these devices cost-effective) and how these factors influence their willingness to use these devices. By integrating these constructs, the UTAUT2 model provides a comprehensive framework for systematically analyzing the acceptance behavior of individual older adults users. Thus, it serves as a critical theoretical foundation for exploring the behavioral intentions and influencing factors of smart wearable devices.

However, given the high propensity of cognitive impairment problems in the older adults, and given also this demographic’s past experience, and social backgrounds (23), it is essential to consider individual differences and their role in the technology adoption process when applying the UTAUT2 model. The Technology Readiness Index (TRI) is a critical factor that reflects individuals’ positive or negative beliefs about technology and plays a significant role in shaping their perceptions, intentions, and behaviors toward new technology (24). While TRI has been extensively studied in predicting new technology acceptance (25, 26), its impact on the use of smart wearable devices by the older adults has not yet been thoroughly examined. Specifically, further exploration is needed on how the older adult’s positive or negative personality traits influence performance expectancy and effort expectancy in the context of smart wearable devices. Additionally, digital health literacy is a crucial skill that enables patients to effectively use technology for disease management and improve public health services (27, 28). In smart older adults care systems, older adults individuals can monitor and transmit health information and access older adults care services through smart wearable devices. This process requires the older adults to master digital media skills and use these tools and information correctly according to their health needs. Low digital health literacy is a significant barrier to adopting and effectively using digital health technologies among the older adults (29). Therefore, exploring the role of digital health literacy in the adoption of wearable devices by the older adults is crucial. Although research on digital health literacy among the older adults is still in its early stages (30), this study will examine how digital health literacy, in conjunction with the UTAUT2 model and TRI, influences the acceptance and adoption of smart wearable devices by the older adults. By incorporating TRI as an antecedent in the UTAUT2 model, we can better understand how personality traits influence older adults users’ perceptions of the usefulness and ease of use of new technologies. Including digital health literacy in the theoretical model not only enhances the model’s explanatory power but also guides the design and promotion strategies of smart wearable devices to better align with the needs and preferences of older adults users.

Based on the above background, this study proposes the following specific research questions:

1. What factors influence older adults users’ intention to adopt smart wearable devices?
2. How does incorporating the TRI structure into the UTAUT2 model enhance its explanatory power in the context of older adults users adopting smart wearable devices?
3. How does the digital health literacy of older adults users impact their willingness to accept smart wearable devices?

The remainder of this paper is organized as follows. First, in Chapter 2, we explore the theoretical background by integrating the UTAUT2 model with TRI and incorporating digital health literacy to create a comprehensive framework, laying the theoretical foundation for this study. Next, we introduce the theoretical model and research hypotheses, followed by a detailed description of the research methods and analysis results. Based on these findings, we discuss the implications and provide limitations and suggestions for future research. This study identifies the factors influencing older adults individuals’ willingness to adopt smart wearable devices. This not only facilitates the implementation of smart older adults care but also offers theoretical and practical guidance for the promotion and application of smart older adults care technology. It helps mitigate the medical burden and pressure on the social medical security system caused by an aging population, providing support for the older adults in society, particularly for empty-nest seniors.

## 2 Theoretical basis and research hypotheses

### 2.1 UTAUT2

The UTAUT model, introduced by Venkatesh et al. (20), includes four key variables: performance expectancy, effort expectancy, social influence, and facilitating conditions. It aims to explain individuals’ acceptance and use of new technology. Recognizing some limitations in its application, Venkatesh et al. (21) developed the UTAUT2 model, adding three dimensions: hedonic motivation, price value, and habit, while considering age, gender, and experience as key moderating variables. The UTAUT2 model significantly improved the explanatory power for predicting individual users’ technology acceptance and use, raising the prediction accuracy of acceptance intention to 74%. Furthermore, the UTAUT2 model has been successfully applied across various research fields, including e-commerce (31), banking (32), virtual try-on technology (24), and personal cloud services (33). Studies by Huang (34) and Macedo (35) have validated the model’s applicability and reliability for older adults users and smart devices through extensive literature reviews, reinforcing this study’s theoretical foundation. Given the UTAUT2 model’s wide applicability and predictive accuracy, this study will use it to explore the acceptance of smart wearable devices among the older adults.

### 2.2 TRI

Technology Readiness (TR) reflects an individual’s inclination and beliefs toward adopting innovative technology products or services. The Technology Readiness Index (TRI) is a psychological scale designed to measure a person’s willingness to accept and use new technology (36). TRI assesses technological readiness by evaluating four key personality traits: optimism, innovativeness, discomfort, and insecurity. Optimism and innovativeness act as motivators, encouraging positive acceptance and use of new technology, while discomfort and insecurity serve as barriers, reducing the likelihood of adoption (37). Since its development, the TRI model has been widely used to study acceptance and adoption of new technologies, products, platforms, and service models, demonstrating broad applicability and predictive capability (33, 38). TRI considers both subjective feelings toward new technology and the willingness to adopt it, highlighting the importance of users’ readiness and emotional preferences in the technology acceptance process. The TRI model has been utilized to study the attitudes of older adults individuals toward technology across different countries and regions. For instance, Shirahada (39) explored the impact of personal beliefs on the attitudes of older adults individuals in Japan and the UK toward using online public services. Sell (40) examined the tendency of older adults individuals in Finland to adopt and use technology for personal purposes. Ramírez-Correa et al. (41) investigated the adoption of social networking sites by the older adults. Additionally, Dash and Mohanty (42) identified key factors influencing the willingness of the older adults to adopt and use mobile health (m-health) technologies. Given the unique challenges and complexities of technology acceptance among the older adults, TRI provides a comprehensive framework to better understand the factors that promote or hinder their acceptance of smart wearable technology. By analyzing optimism, innovativeness, discomfort, and insecurity, we can uncover the attitudes and beliefs of older adults individuals toward smart wearable technology, as well as their psychological barriers and motivations. This analysis provides a theoretical basis for designing more effective strategies.

### 2.3 Digital health literacy

Norman and Skinner (43) first introduced the concept of eHealth literacy (eHL), which refers to an individual’s ability to seek, find, understand, and evaluate health information from electronic sources and apply this knowledge to address health problems. They later noted that as new technologies emerge and personal, social, and environmental contexts change, the ways health knowledge is disseminated will evolve (44). Recognizing this, scholars proposed a new concept: digital health literacy (DHL). DHL extends the concept of health literacy to include the ability to acquire, process, and understand health information and services using digital technologies, thereby promoting and improving individual and collective health through effective health Decision-making (45). Compared to eHealth literacy, DHL emphasizes updated methods for acquiring health information, accurately understanding, disseminating, and communicating health information, and focusing on privacy protection. For the older adults, high levels of digital health literacy are especially important when using internet technology for health management, as it helps them better assess disease risks and understand health information. However, studies show that the overall eHealth literacy of the older adults in China is low, with many facing difficulties in acquiring, understanding, and applying health information (46, 47). This situation is a major barrier to improving self-health management among the older adults. In China, 95.09% of the older adults believe learning to use the internet is very necessary after the pandemic, and 93.36% believe they can learn to use a smartphone for internet access (48), indicating a strong willingness to use digital technology. Among the older adults, a lack of digital health literacy not only affects their acceptance and use of smart wearable devices but also impacts their health decision-making and management effectiveness. Therefore, this study will explore the impact of digital health literacy on the older adult’s acceptance of smart wearable devices.

### 2.4 Theoretical model of this study: combining UTAUT2 and TRI

To explore technology acceptance among the older adults, particularly within China’s specific socio-cultural context, this paper analyzes previous research findings. Chen et al. (49) identified performance expectancy, perceived cost, and hedonic motivation as key factors influencing Chinese older adults people’s acceptance of smart wearable devices. Li et al. (50) highlighted the significant impact of perceived usefulness, compatibility, facilitating conditions, and self-reported health status on the willingness of Chinese older adults to use smart wearable systems for health monitoring. Peng et al. (51) emphasized the user needs for wearable healthcare technology in China’s aging population, particularly regarding healthcare, data privacy, security, and entertainment. These studies indicate that various factors influence older adults people’s acceptance of smart wearable devices. However, current research lacks a comprehensive understanding of how external factors, intrinsic psychological traits, and the ability to acquire, process, and understand health information affect their acceptance behavior.

This study aims to provide a comprehensive perspective on understanding the complexity of older adults people’s acceptance of new technology by analyzing the impact of the TRI model on performance expectancy and effort expectancy, combined with the UTAUT2 model and digital health literacy. In existing research on the adoption of smart technology by the older adults, such as the studies by Macedo (35) and Li et al. (52), the UTAUT2 model has been used. This model has significant advantages due to its effective predictive ability and high explanatory power for technology use (53). However, the UTAUT2 model mainly focuses on evaluating external social and functional factors, with less attention given to the internal psychological states and individual differences of users (24). Particularly, the UTAUT2 model is insufficient in explaining the influence of users’ personality traits on technology adoption. In contrast, the TRI model emphasizes psychological traits such as optimism, innovativeness, discomfort, and insecurity, primarily focusing on the psychological attitudes of individuals toward new technology. However, the TRI model’s neglect of external conditions and other objective factors makes its explanation of the technology adoption process less comprehensive. Therefore, to compensate for these shortcomings, we propose integrating the TRI model into the UTAUT2 model, thereby examining the adoption of technology by older adults users from a more comprehensive perspective. This integrated approach not only considers social and functional factors but also includes individual psychological factors, thereby providing a more thorough understanding of the acceptance and use of smart technology by older adults users.

Expanding technology acceptance models can involve introducing external variables as antecedents to perceived ease of use and perceived usefulness and adding mediating variables such as attitudes and behavioral intentions to jointly explain users’ acceptance behavior (103). Kim and Chiu (38) and Kampa (54) emphasized the importance of combining perceived usefulness and perceived ease of use with the TRI model in explaining technology acceptance. Performance expectancy and effort expectancy, concepts from the TAM model, originate from perceived usefulness and perceived ease of use. The psychological traits in TRI can directly influence performance expectancy and effort expectancy in UTAUT2. Previous studies, such as those by Leong et al. (104), Alsyouf and Ishak (105), and Sharma and Gera (26), have confirmed the relationships between these variables. They indicate that individual psychological traits mediate the relationship between technology acceptance through performance expectancy and effort expectancy. Considering these variables, the integration of UTAUT2 and TRI can provide a richer analytical framework. This multi-dimensional approach helps better understand how older adults users’ psychological traits influence their acceptance of smart wearable devices. In this framework, digital health literacy is a key factor in how the older adults evaluate and accept smart wearable devices. It represents users’ ability to understand health information and adopt digital tools, which is crucial for improving their acceptance of new technology. Incorporating digital health literacy into the integration of UTAUT2 and TRI can help us better understand how the older adults process and evaluate information about smart wearable technology, thereby influencing their acceptance decisions.

Considering the low adoption rate of smart wearable technology among the older adults and the lack of established habits with this technology, we decided to exclude the “habit” variable. Furthermore, given the specific characteristics of the older adults population, slight variations in age, gender, and experience may be less significant compared to other factors such as health literacy and technological readiness. Although these factors might influence technology acceptance in some contexts, they do not seem to be critical moderating variables in this study. Therefore, we chose to remove these moderating variables from the UTAUT2 model. By integrating these theoretical insights and carefully selecting variables, we aim to develop a comprehensive theoretical model to better understand the acceptance behavior of the older adults toward smart wearable devices.

## 3 Conceptual model and hypotheses

Performance expectancy is defined as the extent to which an individual believes that using a system will enhance their work performance (20). In this study, it refers to the older adults users’ expectations of the benefits brought by smart wearable device technology. When users anticipate that technology will enhance their performance or productivity, they are more likely to adopt and utilize it (55). Users’ confidence in the ability of smart wearable devices to enhance healthcare efficiency is a critical factor in their adoption of the technology (73). Numerous studies have demonstrated that positive expectations of new technology can significantly and positively influence individual usage behavior (11, 56, 57). Elderly individuals perceive that smart wearable devices can significantly improve their health management effectiveness, thereby increasing their willingness to use these devices. Based on the above analysis, this paper hypothesizes:

*H1*: Performance expectancy has a significant positive effect on older adults users’ willingness to use smart wearable devices.

Effort expectancy refers to an individual’s perception of the effort required to use new technology. In this study, it refers to the older adult’s perception of the ease or difficulty in effectively learning and operating smart wearable device technology. Within the TAM framework, perceived ease of use is a key factor influencing users’ willingness to accept and use new technology (21). The importance of effort expectancy has been validated in various research areas. For instance, in studying factors influencing students’ continued use of online courses (MOOCs), effort expectancy demonstrated a positive effect (58). Similarly, effort expectancy has been found to directly and positively affect higher education students’ intentions to use ChatGPT (56). This suggests that when users perceive technology as easy to learn and use, they are more likely to adopt it. Furthermore, the perceived effort required is influenced by factors such as user interface design, ease of use, the complexity of the technology needed to complete tasks, and users’ perceptions of interactive operation difficulty (106, 107). These factors collectively influence users’ effort expectancy, thereby affecting their acceptance of technology. For the older adults demographic, smart wearable devices that are easier to learn and operate are more likely to be adopted. Therefore, this study hypothesizes:

*H2*: Effort expectancy has a significant positive effect on older adults users’ willingness to use smart wearable devices.

Facilitating conditions refer to the technical infrastructure that supports the use of systems, focusing on the impact of environmental factors on individuals’ behavioral intentions and actions. In this study, it pertains to the extent to which older adults individuals accept the availability of technical support and resources for smart wearable devices. The presence of such conditions can boost the confidence and self-efficacy of the older adults in using these devices. When support and resources are readily accessible, older adults users are more likely to feel capable of overcoming any challenges or difficulties associated with the device usage. This increased confidence can positively influence their intention to continue using these devices. The significance of facilitating conditions has been highlighted in numerous studies. For example, Wut et al. (59) found that conditions such as fast internet, computing facilities, and mobile-compatible software enhance students’ willingness to engage with online learning platforms. Similarly, research by Huang et al. (60) and Kalınkara and Özdemir (61) demonstrates that facilitating conditions significantly affect individuals’ acceptance of new technologies. Specifically for the older adults, Li et al. (50) revealed that facilitating conditions significantly impact the willingness of Chinese older adults to use smart wearable devices for health monitoring. Unlike younger users, the older adults May face physical limitations or lower technical literacy. Thus, user-friendly interfaces, clear instructions, and robust technical support are crucial to improving the accessibility and usability of smart wearable devices. When the older adults perceive that necessary organizational and technical support is available, they are more likely to persist in using these devices. Therefore, the following hypothesis is proposed:

*H3*: Facilitating conditions have a significant positive effect on older adults users’ willingness to use smart wearable devices.

The impact of social factors and others’ opinions on individual technology adoption becomes particularly significant when influential individuals or groups in social networks recommend a technology (21, 62). This study examines the extent to which the acceptance behavior of the older adults toward smart wearable devices is influenced by family, friends, and peers. Huang et al. (60) found that the willingness of Chinese older adults to adopt smart older adults care is influenced by personal, family, and health factors, with social influence being crucial. Peer recommendations and endorsements (33), the influence of social networks (53), and family members’ involvement and support (108) are key factors that promote individual acceptance of new technology. Elderly individuals tend to conform to social norms and expectations. If significant others in their lives demonstrate positive attitudes and beliefs toward using smart wearable devices, the older adults are more likely to perceive these devices as indispensable and consequently use them. Therefore, the following hypothesis is proposed:

*H4*: Social influence has a significant positive effect on older adults users’ willingness to use smart wearable devices.

Hedonic motivation refers to the pleasure or enjoyment an individual derives from interacting with technology (21). For older adults users, deriving enjoyment and satisfaction from using smart wearable devices can enhance their willingness to adopt these technologies. This concept has been shown to significantly influence technology acceptance and usage willingness in various fields. For instance, Chen et al. (49) indicated that the hedonic motivation of information systems and services is a primary driver of user perception. In medical education, Azizi et al. (63) found that hedonic motivation significantly affects students’ acceptance and use of hybrid learning methods. Vinerean et al. (64) proposed that hedonic motivation is the strongest predictor of consumers’ intentions to continue using mobile commerce. Based on the above discussion, the following hypothesis is proposed:

*H5*: Hedonic motivation has a significant positive effect on older adults users’ willingness to use smart wearable devices.

Venkatesh et al. (21) define price value as the rational balance between the expected benefits of using a system and its financial costs. In this study, it examines how the older adult’s acceptance of smart wearable devices is influenced by their perceived cost-effectiveness. Elderly customers are more price-sensitive than younger ones (65) and have different criteria for evaluating the benefits of technology. This phenomenon is evident in Kaur and Arora (66) study on online banking service usage, which found a strong positive relationship between users’ behavioral intention and price value. Similarly, Li and Fan (67) demonstrated that price value is a major factor in the older adult’s acceptance of augmented reality in tourism scenarios. Chinese older adults users particularly focus on the cost–benefit ratio when considering smart wearable devices. Their willingness to use such devices increases only after they clearly perceive the actual benefits. Based on this analysis, the following hypothesis is proposed:

*H6*: Price value has a significant positive effect on older adults users’ willingness to use smart wearable devices.

In recent years, with the increasingly widespread application of smart wearable devices in health monitoring and management, research on how to improve the willingness of the older adults to use these devices has become particularly important. Existing studies indicate that the older adults generally have low digital health literacy (30). This low literacy not only increases the difficulty of interaction but also reduces their willingness to continuously engage in e-health (68). However, research has also found that older adults individuals with a positive attitude toward health knowledge (69), an interest in digital technology, and confidence in managing health through digital devices (70, 71) often rate their e-health literacy higher. Elderly users with high digital health literacy are more likely to understand and use the health information and services provided by smart wearable devices, making them more willing to adopt these devices to improve their health management. Effective interventions to improve the digital health literacy of the older adults can significantly improve their health status and promote healthy aging (72, 73). Users with high digital health literacy are better able to understand and appreciate the performance advantages of the devices, enhancing the impact of performance expectancy on behavioral intention. At the same time, they can better master the usage of the devices, reducing perceived usage difficulty, thereby increasing behavioral intention. Based on the above analysis, this paper proposes the following hypotheses:

*H7*: Digital health literacy has a significant positive impact on the willingness of older adults users to use smart wearable devices.

*H7a*: Digital health literacy positively moderates the relationship between performance expectancy and behavioral intention.

*H7b*: Digital health literacy positively moderates the relationship between effort expectancy and behavioral intention.

Optimism reflects an individual’s positive attitude and expectations toward new technology, leading optimistic individuals to focus less on potential negative impacts and making them more likely to accept new technology (36). This study suggests that optimistic older adults individuals are more likely to expect positive outcomes from smart wearable devices. Within the Technology Acceptance Model framework, optimism has been extensively studied and shown to positively influence technology acceptance and behavioral intentions. Optimistic older adults users are more likely to believe that smart wearable devices will enhance their quality of life, thereby promoting the acceptance of these devices. For example, Qasem (24) found that optimism significantly impacts performance expectancy regarding customers’ willingness to use virtual try-on technology for online clothing purchases. Similarly, Kim and Park (74) reported a positive correlation between optimism and perceived usefulness, while Kilani et al. (11) demonstrated that optimism strongly influences performance expectancy and effort expectancy in their study on Malaysians’ readiness to use e-wallets. Performance expectancy and effort expectancy are key predictors of older adults individuals’ willingness to use information and communication technology (35). These expectancies serve as intermediaries linking optimism to actual technology behavioral intentions. Optimism initially affects performance expectancy and effort expectancy, which subsequently influence behavioral intentions. This sequence suggests that performance expectancy and effort expectancy mediate the relationship between optimism and behavioral intentions. Based on this analysis, the following hypotheses are proposed:

*H8a*: Optimism positively affects performance expectancy for older adults users using smart wearable devices.

*H9a*: Optimism positively affects effort expectancy for older adults users using smart wearable devices.

*H10a*: Performance expectancy significantly mediates the relationship between optimism and behavioral intention.

*H11a*: Effort expectancy significantly mediates the relationship between optimism and behavioral intention.

Innovativeness is defined as an individual’s willingness to be a technology pioneer and try new technologies (36). This study represents the willingness of older adults individuals to try smart wearable devices. Elderly users with high innovativeness are open to adopting new technologies and are more likely to accept smart wearable devices due to their tendency to actively explore new things. Research indicates that innovativeness is a key predictor of the willingness to use innovative technology (75). Numerous studies have shown that innovativeness positively affects technology acceptance and usage (76), with individuals who possess strong innovative capabilities and awareness being more likely to embrace new technologies. Additionally, Walczuch et al. (77) found that innovativeness not only relates to technology adoption willingness but also influences individuals’ assessments of the usefulness and ease of use of new technologies. In the context of mobile fitness apps, Cui (78) found that innovativeness significantly impacts perceived ease of use and perceived usefulness. Li et al. (11) demonstrated that innovativeness positively influences older adults users’ performance expectancy in internet health management services. Noor et al. (79) showed that innovativeness significantly moderates the relationship between performance expectancy and effort expectancy, impacting users’ intentions to use mobile government services. Innovativeness significantly affects performance expectancy and effort expectancy, which in turn influence users’ willingness to use wearable medical devices (80). Based on this analysis, the following hypotheses are proposed:

*H8b*: Innovativeness positively affects performance expectancy for older adults users using smart wearable devices.

*H9b*: Innovativeness positively affects effort expectancy for older adults users using smart wearable devices.

*H10b*: Performance expectancy significantly mediates the relationship between innovativeness and behavioral intention.

*H11b*: Effort expectancy significantly mediates the relationship between innovativeness and behavioral intention.

In Parasuraman’s (36) study, discomfort refers to the unease or lack of confidence individuals experience when using new technology. This study addresses the older adult’s personal concerns regarding the use of smart wearable technology. Discomfort associated with technology can diminish their confidence and self-efficacy in using smart wearable devices. Walczuch et al. (77) found that higher levels of discomfort with new technologies or business models significantly lower an individual’s attitudes toward use, thereby affecting their willingness to adopt the product. Oh et al. (81) further emphasized how discomfort reduces individuals’ willingness to use new technology. Research by Hackbarth et al. (82) and Igbaria et al. (83) also demonstrated that anxiety about adopting new technology negatively impacts perceived ease of use and the assumed benefits. Feeling a lack of competence in handling a new technology may especially lead individuals to reject adopting them. For older adults users, unfamiliarity with or lack of confidence in operating technology can lower their performance expectations and anticipated effort, thereby hindering the acceptance and use of smart wearable devices. Based on this, the study hypothesizes:

*H8c*: Discomfort negatively affects performance expectancy for older adults users using smart wearable devices.

*H9c*: Discomfort negatively affects effort expectancy for older adults users using smart wearable devices.

*H10c*: Performance expectancy significantly mediates the relationship between discomfort and behavioral intention.

*H11c*: Effort expectancy significantly mediates the relationship between discomfort and behavioral intentions.

Insecurity refers to concerns about potential negative consequences of using technology (36). This study examines the older adult’s subjective assessment of the potential risks associated with using smart wearable technology. Wearable devices typically require users to share personal information and related data to provide personalized insights and enhance the user experience. When sharing this information, users may carefully weigh the trade-offs between potential privacy risks and the benefits involved. Lu et al. (84) confirmed that insecurity negatively impacts perceived ease of use and perceived usefulness of technology. For older adults users, concerns about the safety and privacy of new technology may reduce their performance expectancy and effort expectancy when using these devices. Security dimensions, such as protecting private information and ensuring risk-free payments, are positively related to user trust (109). McCloskey (85) emphasized that security and privacy issues are major barriers to technology acceptance. Dong et al. (86) further confirmed that a lack of security leads to elevated concerns during the adoption of new technology. Walczuch et al. (77) found that insecurity negatively affects the perceived usefulness and perceived ease of use among financial services users, leading to friction in the adoption of new technologies. Peng et al. (51) identified the need for security and data privacy among the aging population in China regarding wearable healthcare technology. Based on this analysis, the study hypothesizes:

*H8d*: Insecurity negatively affects performance expectancy for older adults users using smart wearable devices.

*H9d*: Insecurity negatively affects effort expectancy for older adults users using smart wearable devices.

*H10d*: Performance expectancy significantly mediates the relationship between insecurity and behavioral intention.

*H11d*: Effort expectancy significantly mediates the relationship between insecurity and behavioral intention.

**Figure 1:**
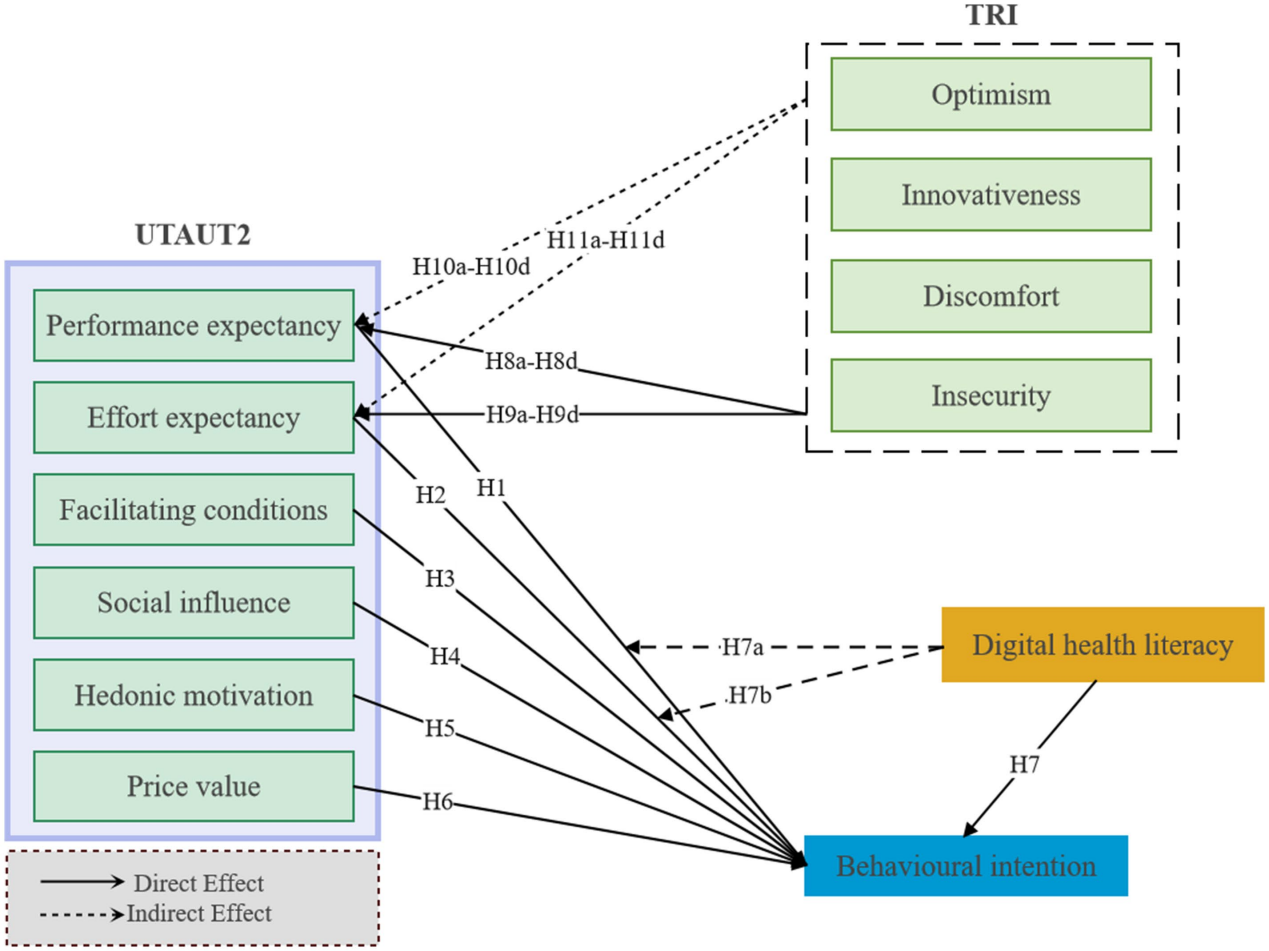
Research model.

Based on these hypotheses, the research framework of this paper is shown in Figure 1.

## 4 Research method

### 4.1 Design of the questionnaire

The survey questionnaire in this study consists of two parts: demographic variables and variable measurements. The first part includes demographic variables such as gender, age group, education level, and monthly income level. The second part involves variable measurements, which use a 5-point Likert scale (1 = “strongly disagree”; 5 = “strongly agree”) to assess each variable in the study. The questionnaire includes a set of measurement items based on the aforementioned variables. These items are adapted from established scales in relevant domestic and international research, with modifications to fit the actual situation of older adults users, ensuring the reliability and validity of the questionnaire. Details of the variable measurement items and their sources are provided in Table 1.

**Table 1:** Subjects and resources for variable measurement.

Main category	Category	Source
Performance expectancy	PE1 I find that using smart wearable devices makes my daily life more convenient. PE2 Smart wearable devices improve work efficiency and help complete tasks faster. PE3 I find smart wearable devices very useful in daily life.	(20, 21, 95)
Effort expectancy	EE1 Learning to use smart wearable devices is easy for me. EE2 I find smart wearable devices easy to understand and operate. EE3 I find using smart wearable devices simple. EE4 It is easy for me to become proficient in using smart wearable devices.	
Social influence	SI1 People around me support my use of smart wearable devices. SI2 People important to me believe I should use smart wearable devices. SI3 People around me think I should use smart wearable devices.	
Facilitating conditions	FC1 I have the necessary knowledge and skills to use smart wearable devices. FC2 I have the resources needed to use smart wearable devices, such as a smartphone and internet access. FC3 Smart wearable devices are compatible with other systems, making them convenient for daily use. FC4 When I encounter difficulties using smart wearable devices, I can receive help from others.	
Hedonic motivation	HM1 Using smart wearable devices is very interesting. HM2 Using smart wearable devices is very pleasant. HM3 Using smart wearable devices is very enjoyable.	
Price value	PV1 The price cost required for smart wearable devices is reasonable. PV2 Smart wearable devices are worth the money. PV3 Compared to the cost required to use smart wearable devices, the satisfaction gained is greater.	
Optimism	OP1 Smart wearable devices help me achieve a higher quality of life. OP2 Smart wearable devices give me more freedom in choosing locations. OP3 Smart wearable devices enable me to better control my daily life. OP4 Smart wearable devices make my life more productive.	(36, 96)
Innovativeness	INN1 People around me ask for my advice about new smart wearable device products/services. INN2 Generally, I am one of the first among my friends to use smart wearable device products. INN3 Without others’ help, I can usually quickly familiarize myself with smart wearable device products/services. INN4 In areas that interest me, I keep up with the latest technological developments.	
Discomfort	DIS1 When seeking help from others for smart wearable device support, I sometimes feel like I am being controlled. DIS2 Online support for smart wearable devices does not adequately clarify my issues. DIS3 Sometimes I feel that smart wearable device systems are too complex for general use. DIS4 The user manuals for smart wearable device products/services are not easy to understand.	
Insecurity	INS1 In my daily life, I feel overly dependent on some smart wearable device products/services. INS2 I believe smart wearable devices are not secure and worry about my privacy. INS3 I am concerned that the information from smart wearable devices may be inaccurate.	
Digital health literacy	DHL1 I am able to use smart wearable devices to find the information I need. DHL2 I know how to find useful health resources on smart wearable devices. DHL3 I know how to use the health information from smart wearable devices to help myself. DHL4 I am confident in making health-related decisions based on information from smart wearable devices. DHL5 I have the skills to distinguish between good and bad information from smart wearable device resources.	(30, 97, 98, 100, 101)
Behavioral intention	BI1 I frequently use smart wearable devices. BI2 I will share my experiences with smart wearable devices with others. BI3 I will continue to use smart wearable devices.	(99)

### 4.2 Data collection

The data collection process comprised two main stages: preliminary investigation and formal investigation. Preliminary Investigation: Initially, three peer experts were invited to evaluate the draft questionnaire, focusing on its structure and content clarity. Based on their feedback, we conducted a pre-test with 15 older adults individuals who used smart wearable devices, ensuring a diverse sample in terms of gender and age (over 60 years old). The pre-test aimed to evaluate the questionnaire’s comprehensibility and completion time. A significant challenge identified was the difficulty some older adults individuals had in understanding certain technical terms. To address this, we revised and enhanced the questionnaire content for better clarity. To ensure respondent cooperation, the questionnaire was designed to be concise and clear, with carefully controlled question lengths to minimize invalid or perfunctory responses. This process resulted in the final version of the questionnaire. This study evaluates commercially available, non-customized everyday smart wearable devices, directly marketed to consumers without any customization. By selecting non-customized devices from mainstream brands, we aim to understand the actual user experience and acceptance among older adults users. An in-depth evaluation of these non-customized devices will help accurately identify the practical challenges and obstacles faced in promoting these devices to older adults users. Formal Investigation: During this stage, we first used the snowball method to distribute the online survey questionnaire in community group chats, allowing older adults individuals or their children to complete it based on the older adult’s actual conditions. Additionally, we distributed paper questionnaires in crowded areas such as subway stations, urban centers, and communities. For older adults respondents with hearing or vision impairments, investigators provided detailed oral explanations of each question and option. If respondents had difficulty making choices, investigators assisted them patiently. To ensure the validity of the collected data, we excluded questionnaires with missing answers, insufficient completion times, or invalid information. Ultimately, 389 valid questionnaires were included in the data analysis. The demographic characteristics of the respondents are detailed in Table 2.

**Table 2:** Sample’s demographic profile.

Category	Option	Frequency	Percentage (%)
Gender	Male	227	58.4
	Female	162	41.6
Age range	60–65 years	187	48.1
	66–70 years	115	29.6
	71–75 years	58	14.9
	75 years and above	29	7.4
Education level	Junior high school	175	45.0
	Elementary school or below	154	39.6
	High school or above	60	15.4
Monthly income	Below 3,000 RMB	117	30.1
	3,000–5,000 RMB	176	45.2
	5,000–7,000 RMB	76	19.5
	Above 7,000 RMB	20	5.2
Total		389	100

### 4.3 Data analysis

In this study, SPSS and AMOS software were used to assess the reliability and validity of the scales. Using the eigenvalue method, seven common factors were extracted, with factor loadings above 0.7 for each factor, indicating that these seven variables are effectively measured by the respective indicators. The Cronbach’s alpha coefficients for all variables exceeded the standard value of 0.7, demonstrating good internal consistency among the items within each variable. The composite reliability (CR) and average variance extracted (AVE) values exceeded the thresholds of 0.7 and 0.5, respectively, indicating good convergent validity for the model (87), as shown in Table 3. If the square root of the AVE of each construct is greater than the correlation coefficients between constructs, the model demonstrates discriminant validity. In this study, the square roots of the AVEs on the diagonal were higher than the correlation coefficients off the diagonal, indicating good discriminant validity among the latent variables, as shown in Table 4.

**Table 3:** Summary results of convergent validity and reliability.

Main category	Observed variable	Standardized loading	Cronbach’s a	CR	AVE
Performance expectancy	PE1 PE2 PE3	0.824 0.826 0.814	0.907	0.908	0.768
Effort expectancy	EE1 EE2 EE3 EE4	0.824 0.771 0.807 0.790	0.918	0.919	0.741
Social influence	SI1 SI2 SI3	0.850 0.856 0.812	0.914	0.916	0.786
Facilitating conditions	FC1 FC2 FC3 FC4	0.828 0.811 0.821 0.776	0.925	0.926	0.759
Hedonic motivation	HM1 HM2 HM3	0.800 0.812 0.806	0.903	0.904	0.758
Price value	PV1 PV2 PV3	0.774 0.804 0.803	0.904	0.905	0.760
Optimism	OP1 OP2 OP3 OP4	0.805 0.811 0.809 0.793	0.933	0.934	0.778
Innovativeness	INN1 INN2 INN3 INN4	0.858 0.790 0.838 0.847	0.926	0.927	0.760
Discomfort	DIS1 DIS2 DIS3 DIS4	−0.852 −0.786 −0.810 −0.822	0.935	0.936	0.785
Insecurity	INS1 INS2 INS3 INS4	−0.842 −0.815 −0.818 −0.806	0.934	0.935	0.783
Digital health literacy	DHL1 DHL2 DHL3 DHL4 DHL5	0.793 0.775 0.794 0.786 0.786	0.929	0.929	0.725
Behavioral intention	BI1 BI2 BI3	0.951 0.902 0.898	0.906	0.910	0.772

CR, composite reliability; AVE, average variance extracted.

**Table 4:** Correlation matrix and discriminant validity.

	PE	EE	SI	FC	HM	PV	OP	INN	DIS	INS	DHL	BI
PE	**0.876**											
EE	0.424	**0.861**										
SI	0.343	0.378	**0.886**									
FC	0.425	0.407	0.414	**0.871**								
HM	0.407	0.430	0.344	0.423	**0.871**							
PV	0.398	0.486	0.401	0.477	0.414	**0.872**						
OP	0.425	0.468	0.422	0.418	0.438	0.501	**0.882**					
INN	0.409	0.376	0.396	0.39	0.449	0.377	0.372	**0.872**				
DIS	−0.457	−0.461	−0.360	−0.392	−0.415	−0.409	−0.479	−0.388	**0.886**			
INS	−0.382	−0.396	−0.366	−0.44	−0.444	−0.441	−0.436	−0.366	0.495	**0.885**		
DHL	0.385	0.508	0.410	0.496	0.454	0.461	0.501	0.388	−0.416	−0.469	**0.851**	
BI	0.495	0.560	0.486	0.546	0.516	0.562	0.574	0.498	−0.530	−0.514	0.573	**0.878**

The square root of the average variance extracted for a construct is shown by scores that are bolded.

### 4.4 Structural modeling and hypothesis testing

This study used AMOS software to evaluate the structural equation model and test its overall fit. The model’s fit indices are as follows: CMIN/DF = 1.361, GFI = 0.93, NFI = 0.93, CFI = 0.98, and RMSEA = 0.03. These values fall within the acceptable range (88), indicating a good fit. Additionally, AMOS 26.0 was used to test the hypothesized paths between latent variables in the theoretical model. The path coefficients and the results of the corresponding hypothesis tests are presented in Table 5.

**Table 5:** Hypotheses testing results.

Factor (latent variable)	→	Analysis item (observed variable)	Non-standardized coefficient	Standardized coefficient	S.E	C.R.	*p*	Conclusion
PE	→	BI	0.150	0.152	0.041	3.632	***	Accepted
EE	→	BI	0.164	0.154	0.044	3.722	***	Accepted
SI	→	BI	0.131	0.135	0.042	3.150	0.002	Accepted
FC	→	BI	0.142	0.126	0.053	2.666	0.008	Accepted
HM	→	BI	0.169	0.166	0.047	3.593	***	Accepted
PV	→	BI	0.194	0.182	0.052	3.732	***	Accepted
DHL	→	BI	0.193	0.189	0.050	3.831	***	Accepted
OP	→	PE	0.198	0.208	0.054	3.680	***	Accepted
INN	→	PE	0.220	0.218	0.053	4.163	***	Accepted
DIS	→	PE	−0.231	−0.245	0.055	−4.184	***	Accepted
INS	→	PE	−0.097	−0.106	0.052	−1.867	0.062	Rejected
OP	→	EE	0.250	0.282	0.049	5.128	***	Accepted
INN	→	EE	0.135	0.144	0.048	2.844	0.004	Accepted
DIS	→	EE	−0.210	−0.239	0.050	−4.205	***	Accepted
INS	→	EE	−0.099	−0.117	0.047	−2.116	0.034	Accepted

*p < 0.05; **p < 0.01; ***p < 0.001.

**Figure 2:**
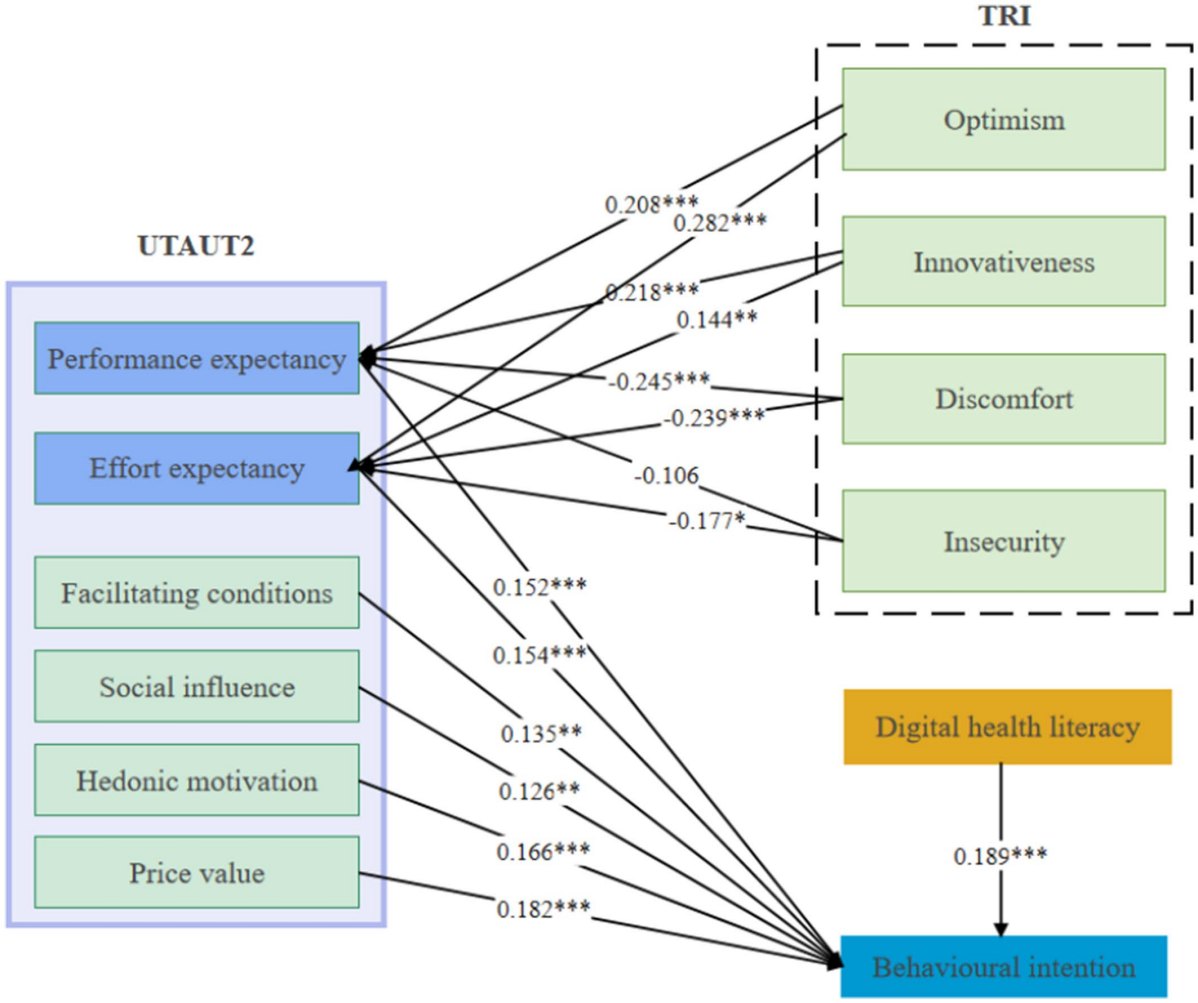
Path coefficients for the research model. *p < 0.05; **p < 0.01; ***p < 0.001.

The specific results are as follows: performance expectancy has a significant positive effect on the intention to use (*β* = 0.152, *p* < 0.001), supporting hypothesis H1. Effort expectancy also shows a significant positive effect on the intention to use (*β* = 0.154, *p* < 0.001), supporting hypothesis H2. Social influence significantly positively affects the intention to use (*β* = 0.135, *p* < 0.05), thus supporting hypothesis H3. Facilitating conditions have a significant positive impact on the intention to use (*β* = 0.126, *p* < 0.05), supporting hypothesis H4. Hedonic motivation significantly positively influences the intention to use (*β* = 0.166, *p* < 0.001), supporting hypothesis H5. Price value significantly positively impacts the intention to use (*β* = 0.182, *p* < 0.001), supporting hypothesis H6. Digital health literacy has a significant positive effect on the intention to use (*β* = 0.189, *p* < 0.001), supporting hypothesis H7. Optimism significantly positively affects performance expectancy (*β* = 0.208, *p* < 0.001), supporting hypothesis H8a. Innovativeness has a significant positive impact on performance expectancy (*β* = 0.218, *p* < 0.001), supporting hypothesis H8b. Discomfort significantly negatively affects performance expectancy (*β* = −0.245, *p* < 0.001), supporting hypothesis H8c. Insecurity shows a negative impact on performance expectancy (*β* = −0.106, *p* = 0.062), which does not support the original hypothesis, hence hypothesis H8d is not supported. Optimism has a significant positive effect on effort expectancy (*β* = 0.282, *p* < 0.001), supporting hypothesis H9a. Innovativeness significantly positively affects effort expectancy (*β* = 0.144, *p* < 0.01), supporting hypothesis H9b. Discomfort has a significant negative impact on effort expectancy (*β* = −0.239, *p* < 0.001), supporting hypothesis H9c. Insecurity significantly negatively affects effort expectancy (*β* = −0.117, *p* < 0.05), supporting hypothesis H9d The detailed results of the hypothesized path analysis are presented in (Figure 2).

### 4.5 Mediating effect test

To explore the internal mechanism through which personality traits of older adults individuals affect their intention to use technology, performance expectancy (PE) and effort expectancy (EE) were introduced as mediating variables in the structural equation model. The mediating effect was tested using the Bootstrap method. Within a 95% confidence interval, a mediating effect is considered significant if the interval estimate does not contain 0; otherwise, it is not significant. The roles of PE and EE as mediators between older adults individuals’ personality traits and their intention to use were analyzed separately. Based on the data in Table 6, the following conclusions can be drawn: Optimism significantly positively affects the intention to use, with a mediating effect value of 0.294. This indicates that optimism positively influences the intention to use through both PE and EE. The mediating effects account for 7.12 and 17.90% of the total effect, respectively, suggesting that PE and EE partially mediate the impact of optimism on the intention to use. Similarly, innovativeness significantly positively impacts the intention to use, with a mediating effect value of 0.241. Innovativeness also positively influences the intention to use through PE and EE, with the mediating effects accounting for 10.34 and 13.98% of the total effect, respectively, indicating partial mediation by PE and EE. Discomfort and insecurity significantly negatively affect the intention to use, with mediating effect values of −0.177 and −0.183, respectively. This shows that discomfort and insecurity negatively influence the intention to use through PE and EE. The mediating effects account for 14.03 and 25.07%, and 6.43 and 13.37% of the total effect, respectively, indicating that PE and EE also partially mediate the impact of discomfort and insecurity on the intention to use. In summary, PE and EE partially mediate the effects of optimism, innovativeness, discomfort, and insecurity on the intention to use. These factors influence older adults users’ PE and EE, thereby affecting their evaluation of the intention to use. To enhance older adults users’ intention to use smart wearable devices, it is beneficial to enhance their optimism and innovativeness while reducing discomfort and insecurity, thereby improving their positive emotions and exploratory experiences.

**Table 6:** Results of mediating effect test.

	Total effect	Mediating effect	Boot SE	*z* value	*p* value	95% BootCI	Direct effect
OP= > PE= > UI	0.294**	0.021	0.011	1.895	0.058	0.004 ~ 0.047	0.220**
OP= > EE= > UI	0.294**	0.053	0.018	2.998	0.003	0.025 ~ 0.093	0.220**
INN= > PE= > UI	0.241**	0.025	0.011	2.232	0.026	0.006 ~ 0.049	0.183**
INN= > EE= > UI	0.241**	0.034	0.014	2.461	0.014	0.010 ~ 0.063	0.183**
DIS= > PE= > UI	−0.177**	−0.025	0.013	−1.9	0.057	−0.056 ~ −0.006	−0.107**
DIS= > EE= > UI	−0.177**	−0.044	0.014	−3.117	0.002	−0.079 ~ −0.023	−0.107**
INS= > PE= > UI	−0.183**	−0.012	0.009	−1.364	0.172	−0.033 ~ −0.000	−0.147**
INS= > EE= > UI	−0.183**	−0.025	0.013	−1.848	0.065	−0.055 ~ −0.004	−0.147**

*p < 0.05; **p < 0.01; ***p < 0.001.

### 4.6 Analysis of the moderating effect of digital health literacy

The regression analysis reveals how digital health literacy (DHL) moderates the impact of performance expectancy (PE) and effort expectancy (EE) on the behavioral intention (BI) to use smart wearable devices among older adults. In PE models, Model 1 shows PE significantly affects BI (*β* = 0.487, *t* = 11.199, *p* < 0.001). Adding DHL in Model 2 shows both PE (*β* = 0.317, *t* = 7.638, *p* < 0.001) and DHL (*β* = 0.439, *t* = 10.643, *p* < 0.001) significantly affect BI, increasing *R*^2^ to 0.416. Model 3 adds the interaction term PE_DHL, indicating a significant negative interaction (*β* = −0.067, *t* = −2.01, *p* < 0.05), meaning higher DHL reduces PE’s positive impact on BI. Similarly, in EE models, Model 1 shows EE significantly affects BI (*β* = 0.605, *t* = 13.295, *p* < 0.001). With DHL in Model 2, both EE (*β* = 0.392, *t* = 8.093, *p* < 0.001) and DHL (*β* = 0.380, *t* = 8.666, *p* < 0.001) significantly affect BI, increasing *R*^2^ to 0.425. Model 3 adds the interaction term EE_DHL, showing a significant negative interaction (*β* = −0.078, *t* = −2.111, *p* < 0.05), meaning higher DHL reduces EE’s positive impact on BI. These findings suggest that while PE and EE positively influence the intention to use smart wearable devices, higher levels of DHL weaken these effects. The results of the study are shown in Table 7. Therefore, the results of the study do not support hypotheses H7a, H7b.

**Table 7:** Analysis of the moderating role of DHL results.

Model	Variable	Coefficient	*t*-value	*p*-value	*R* ^2^	Adjusted *R*^2^	Δ*R*^2^	Δ*F*-value
Model 1	PE	0.487	11.199	0.000***	0.245	0.243	0.245	ΔF (1,389) = 125.411, *p* = 0.000***
Model 2	PE	0.317	7.638	0.000***	0.416	0.413	0.416	ΔF (1,386) = 113.264, *p* = 0.000***
	DHL	0.439	10.643	0.000***				
Model 3	PE	0.545	4.509	0.000***	0.422	0.418	0.422	ΔF (1,385) = 142.65, *p* = 0.000***
	DHL	0.674	5.435	0.000***				
	PE*DHL	−0.067	−2.01	0.045**				
Model 1	EE	0.605	13.295	0.000***	0.314	0.312	0.314	ΔF (1,389) = 176.75, *p* = 0.000***
Model 2	EE	0.392	8.093	0.000***	0.425	0.422	0.425	ΔF (1,386) = 75.107, *p* = 0.000***
	DHL	0.38	8.666	0.000***				
Model 3	EE	0.664	4.822	0.000***	0.432	0.427	0.432	ΔF (1,385) = 148.589, *p* = 0.000***
	DHL	0.621	5.078	0.000***				
	EE*DHL	−0.078	−2.111	0.035**				

*p < 0.05; **p < 0.01; ***p < 0.001.

## 5 Conclusion and implications

### 5.1 Conclusion

This study employs the UTAUT2 and TRI models as the primary theoretical frameworks, incorporating previous research on the usage behavior of smart wearable devices to identify influencing factors and construct a research model. By introducing digital health literacy as an innovative factor, the study enhances and deepens the understanding of technology acceptance and adoption theories. The results indicate that performance expectancy, effort expectancy, social influence, facilitating conditions, hedonic motivation, price value, and digital health literacy are positive predictors of older adults’ willingness to use smart wearable devices. These findings reveal the complex factors influencing older adults’ willingness to use these devices, effectively addressing Research Question 1. Previous studies on smart technology primarily focused on the UTAUT2 model, emphasizing external social and functional factors. However, the UTAUT2 model has limitations in explaining the acceptance of smart wearable devices by older users, particularly concerning individual traits. Therefore, this study integrates the TRI framework to enhance the explanatory power of the UTAUT2 model, providing additional insights into attitudes and behavioral intentions. The TRI framework refines the analysis of individual differences by quantifying older users’ scores on optimism, innovativeness, discomfort, and insecurity. The findings show that optimism, innovativeness, and discomfort have a significant positive effect on performance expectancy, while optimism, innovativeness, discomfort, and insecurity positively influence effort expectancy. Performance expectancy and effort expectancy mediate the relationship between personality traits and behavioral intention, providing a comprehensive understanding of older users’ acceptance behavior, thus addressing Research Question 2. Additionally, the results confirm that digital health literacy significantly positively influences older users’ willingness to use smart wearable devices and negatively moderates the relationship between performance expectancy, effort expectancy, and behavioral intention. This indicates that older adults with higher digital health literacy are less dependent on device performance and adapt more easily to new technologies, answering Research Question 3. These findings strongly support the proposed model and warrant further discussion.

This study found that performance expectancy has a significant positive impact on older adults’ willingness to use smart wearable devices. This can be explained by the fact that when older adults individuals believe these devices can significantly enhance service quality and user experience, their willingness to use them increases. This conclusion aligns with the findings of Chen et al. (49), indicating the potential of smart wearable devices to gain acceptance among the older adults. Furthermore, effort expectancy has a significant positive impact on their willingness to use these devices, consistent with the results of Li and Zhao (58). The results indicate that older users’ positive behavioral intentions are primarily driven by performance expectancy and effort expectancy. Smart wearable devices that effectively manage health and offer intuitive interfaces with simple operating instructions can reduce the effort required by users, thereby enhancing both expectancies. As older users find these devices easy to master and beneficial for their health, the reduced burden of learning new technology makes them more willing to adopt and use these devices.

Social influence has also been demonstrated to have a significant positive effect on the willingness of older adults individuals to use smart wearable devices. This finding is consistent with the research by Martinez and McAndrews (108) and further validated by Amnas et al. (53). The increased susceptibility of older adults individuals to external opinions may stem from changes in cognitive function, reduced personal independence, or a heightened need for social interaction. They place significant importance on the opinions of family and friends, which can greatly influence their intentions and behaviors. Encouragement, support, and practical demonstrations from family and friends can effectively alleviate fears and uncertainties about new technologies. Facilitating conditions have a significant positive impact on the willingness of the older adults to use smart wearable devices, aligning with the findings of Kalınkara and Özdemir (61). When older adults individuals feel that they have sufficient resources, knowledge, and technical support, the barriers they face in learning and using new technology are significantly reduced, making them more inclined to adopt and use smart wearable devices.

Additionally, hedonic motivation has a significant positive effect on the willingness of older adults individuals to use smart wearable devices, consistent with the findings of Chen et al. (49). This suggests that hedonic motivation is an important and valuable driver of consumer behavioral intention (64). The high hedonic motivation that older adults users derive from these devices can evoke positive emotions, thereby enhancing their usage behavior. This emphasizes the importance of the devices being enjoyable and engaging to use. Price value also significantly promotes the willingness of older adults individuals to use these devices, in line with the findings of Amnas et al. (53). The basic social security level for older adults individuals in rural China is low, and their budget is limited. Additionally, their consumption concepts are still transitioning, and their consumption habits and perceptions have not yet fully aligned with market offerings. When selecting smart products, most older adults people prioritize “good quality at a low price.” Thus, when they perceive the cost of a device to be justified by its benefits, they are more likely to adopt these technologies. This underscores the importance of considering the cost-effectiveness of devices in pricing and marketing strategies.

This study found that digital health literacy significantly enhances the willingness of older adults users to use smart wearable devices, aligning with the findings of Chen et al. (49). Elderly individuals with higher digital health literacy can better understand and assess health-related information and use it to improve their health behaviors (89). They also tend to have better health outcomes and engage in healthier behaviors, such as disease prevention and management (90). These more capable older adults individuals are more likely to adopt wearable technology that aids in health management and enhances their quality of life. Additionally, research has found that digital health literacy negatively moderates the relationship between performance expectancy, effort expectancy, and the intention to use technology. Elderly users with higher digital health literacy usually possess strong confidence and autonomy in health management and technology application. This group shows higher self-efficacy (91), effectively using the information and functions provided by devices and independently solving problems when they arise. Their ability to compensate for potential device deficiencies with their own knowledge and skills makes them less dependent on specific device performance. Consequently, they are less sensitive to performance expectations than older adults individuals with lower digital health literacy. Therefore, even if performance and effort expectations are not high, they are still willing to use these devices. Similarly, high digital health literacy indicates that older adults users are better at learning and adapting to new technologies. They can quickly master the use of devices, show higher acceptance and flexibility toward new technologies, and continuously enhance their operational skills during use. This enables them to adapt to new devices more quickly and gain a sense of accomplishment from their usage, even if some effort is required. Consequently, effort expectancy has a smaller impact on their intention to use. These findings provide important theoretical support for promoting and applying smart wearable devices among the older adults.

Research indicates that optimism significantly enhances performance expectancy and effort expectancy among older adults users, as noted by Qasem (24). Optimism is not just a psychological trait but a powerful internal motivator. Optimistic older adults users tend to believe that using smart wearable devices can help them better manage their health and improve their quality of life. Individuals with optimistic traits have a positive perception of technology (92). In this context, performance expectancy and effort expectancy serve as mediators, concretizing the positive effects of optimism and demonstrating how optimism translates into actual usage behavior. Optimistic older adults individuals lower their expectations of potential difficulties and are inclined to believe that using smart wearable devices will lead to positive outcomes. This positive expectation encourages them to try and use these devices.

Innovativeness directly positively influences older adults users’ performance expectancy and effort expectancy for smart wearable devices (78, 93). This positive attitude arises from their openness to the potential benefits of the devices and their inclination to explore new features. They believe they can easily learn and operate new technologies, and this increased confidence encourages them to actively explore smart wearable devices. Having a higher degree of innovativeness leads older adults users to possess greater expectations of the benefits that smart wearable devices can provide and boosts their confidence in overcoming usage difficulties. This enhanced confidence and higher expectations, mediated by performance expectancy and effort expectancy, further increase their willingness to use these devices. Performance expectancy and effort expectancy serve as crucial mediators, connecting innovativeness with the intention to use. Moreover, this positive outlook not only helps older adults users better adapt to and utilize smart wearable devices but also enhances their acceptance of technology in health management and daily life.

Insecurity significantly negatively affects effort expectancy but does not significantly impact performance expectancy. Elderly individuals may prioritize the benefits of wearable devices and their potential impact on health and well-being, focusing on whether their basic needs are met rather than deep concerns about privacy and technical performance. They tend to weigh the potential benefits against the risks (94). Elderly users might believe they need to invest more time and effort in learning how to protect personal information and handle potential technical issues, which increases their psychological burden regarding new technologies. This resistance not only undermines their trust in the technical performance but also leads to negative evaluations of smart wearable devices. This mindset also affects the adoption rate of smart wearable devices among the older adults.

Discomfort significantly negatively impacts older adults users’ performance and effort expectancy for smart wearable devices, consistent with the findings of Hackbarth et al. (82). If older adults users find these devices complex and difficult to use, their expectations for health improvement and quality of life enhancement decrease. They may lose confidence in the devices’ effectiveness, leading to reduced dependence and usage. Discomfort raises the learning costs, undermines their confidence in mastering new technology, and causes anxiety about potential operational mistakes. This perceived difficulty in learning to use new devices makes them less willing to adopt smart wearable technology.

### 5.2 Theoretical contributions

This study makes several key contributions. The interest of older adults users in smart wearable devices is crucial for the development of the associated industry. Therefore, this research expands the scope of smart wearable device studies, drawing more attention to the needs and behaviors of this significant user group. This study not only validates the applicability of the UTAUT2 and TRI models across different cultures and age groups but also extends these models to the field of smart wearables. This extension integrates theoretical models from behavioral science and information systems, focusing on individual characteristics to provide a comprehensive understanding of older adults users’ acceptance behaviors. Additionally, this research focuses on older adults users in China, addressing a gap in current studies that predominantly target younger Chinese users. By extending the study to the older adults demographic, we contribute to filling this research void. Examining the technology acceptance behavior of Chinese older adults users enables a better understanding of their specific needs within this cultural context, providing scientific support for localized product design and marketing strategies.

### 5.3 Practical implications

From a practical standpoint, this study provides valuable insights into designing, developing, and marketing smart wearable devices for older adults users. It emphasizes prioritizing the actual needs and ease of use for older adults users. For instance, incorporating emergency medical shortcuts, such as alarm buttons and voice call features, can address accidental injuries and sudden illnesses. Design teams must consider the physiological and psychological challenges older adults users face, such as reduced tactile sensitivity and decreased muscle control, which make fine motor tasks challenging. Therefore, minimizing the effort required to use these devices is crucial. This can be achieved through user-friendly designs, clear instructions, intuitive interfaces, and simplified functionalities. For the older adults market, development and marketing teams should segment the market to identify the specific needs and preferences of different older adults groups accurately. Emphasizing cost-effectiveness in pricing strategies and marketing plans is essential. Companies can involve family members in the digital health education of the older adults, providing necessary support, encouraging users to share their achievements and progress, and related health experiences, thereby motivating other older adults individuals to adopt smart wearable devices. Furthermore, digital health literacy training can be provided through community centers and organizations frequently visited by the older adults. Offering step-by-step guides, illustrated instructions, and video demonstrations can help the older adults gradually learn to use the devices, enhancing their digital health literacy. Developers should consider incorporating goals, challenges, and rewards, such as step challenges and health achievement badges, to increase engagement. In China, older adults individuals often exhibit cautious and conservative usage habits, with a tendency to distrust new technologies like smart wearable devices. Therefore, technology experience activities, demonstrations of specific cases, or related videos can visually showcase the devices’ effectiveness, enhancing their perceived value. Additionally, explaining the safety and privacy protection measures can alleviate safety concerns, increase understanding and trust in the technology, and encourage them to use these devices.

### 5.4 Limitations and future directions

While this study provides significant insights into the adoption and use of smart wearable devices among the older adults in China, it has some limitations. The sample is primarily from China, and although older adults individuals often display similar technology acceptance behaviors, this study does not fully account for cultural and socio-economic differences that may exist in Western countries, which could affect the generalizability of the findings. Future research should explore how to adjust or extend these models to better meet the specific needs of the older adults and fit within the Chinese cultural context. For example, incorporating the impact of cultural values on technology acceptance into the models could be beneficial. Additionally, considering factors such as self-efficacy, perceived risk, and health anxiety could provide a more comprehensive understanding of the motivations and challenges faced by the older adults in adopting smart wearable devices. Including these factors may offer deeper insights into how technology can improve the quality of life for the older adults. Future studies should also expand the sample size by using cross-cultural comparative research methods or mixed-method research designs, including older adults individuals from various regions and cultural backgrounds, to enhance the generalizability and applicability of the findings.

## Data Availability

The original contributions presented in the study are included in the article/supplementary material, further inquiries can be directed to the corresponding author.
